# Physiological and Proteomic Responses of Contrasting Alfalfa (*Medicago sativa* L.) Varieties to PEG-Induced Osmotic Stress

**DOI:** 10.3389/fpls.2018.00242

**Published:** 2018-02-28

**Authors:** Cuimei Zhang, Shangli Shi

**Affiliations:** College of Grassland Science, Key Laboratory of Grassland Ecosystem (Ministry of Education), Pratacultural Engineering Laboratory of Gansu Province, Sino-U.S. Centers for Grazing Land Ecosystem Sustainability, Gansu Agricultural University, Lanzhou, China

**Keywords:** alfalfa, progressive osmotic stress, physiological changes, iTRAQ-based proteomics, drought-responsive protein, roots

## Abstract

Drought severely limits global plant distribution and agricultural production. Elucidating the physiological and molecular mechanisms governing alfalfa stress responses will contribute to the improvement of drought tolerance in leguminous crops. In this study, the physiological and proteomic responses of two alfalfa (*Medicago sativa* L.) varieties contrasting in drought tolerance, Longzhong (drought-tolerant) and Gannong No. 3 (drought-sensitive), were comparatively assayed when seedlings were exposed to -1.2 MPa polyethylene glycol (PEG-6000) treatments for 15 days. The results showed that the levels of proline, malondialdehyde (MDA), hydrogen peroxide (H_2_O_2_), hydroxyl free radical (OH^•^) and superoxide anion free radical (O_2_^•-^) in both varieties were significantly increased, while the root activity, the superoxide dismutase (SOD) and glutathione reductase (GR) activities, and the ratios of reduced/oxidized ascorbate (AsA/DHA) and reduced/oxidized glutathione (GSH/GSSG) were significantly decreased. The soluble protein and soluble sugar contents, the total antioxidant capability (T-AOC) and the activities of peroxidase (POD), catalase (CAT), and ascorbate peroxidase (APX) first increased and then decreased with the increase in treatment days. Under osmotic stress, Longzhong exhibited lower levels of MDA, H_2_O_2_, OH^•^ and O_2_^•-^ but higher levels of SOD, CAT, APX, T-AOC and ratios of AsA/DHA and GSH/GSSG compared with Gannong No.3. Using isobaric tags for relative and absolute quantification (iTRAQ), 142 differentially accumulated proteins (DAPs) were identified from two alfalfa varieties, including 52 proteins (34 up-regulated and 18 down-regulated) in Longzhong, 71 proteins (28 up-regulated and 43 down-regulated) in Gannong No. 3, and 19 proteins (13 up-regulated and 6 down-regulated) shared by both varieties. Most of these DAPs were involved in stress and defense, protein metabolism, transmembrane transport, signal transduction, as well as cell wall and cytoskeleton metabolism. In conclusion, the stronger drought-tolerance of Longzhong was attributed to its higher osmotic adjustment capacity, greater ability to orchestrate its enzymatic and non-enzymatic antioxidant systems and thus avoid great oxidative damage in comparison to Gannong No. 3. Moreover, the involvement of other pathways, including carbohydrate metabolism, ROS detoxification, secondary metabolism, protein processing, ion and water transport, signal transduction, and cell wall adjustment, are important mechanisms for conferring drought tolerance in alfalfa.

## Introduction

Drought is a major abiotic stress that limits the geographic distribution and productivity of crop plants worldwide, resulting in enormous losses of yield (Farooq et al., 2009; Fang and Xiong, 2015; Cao et al., 2017). According to Sivakumar et al. (2005), arid and semi-arid regions make up approximately one-third of the total area worldwide. In the future, more frequent and severe drought in many areas will be intensified by global warming, deforestation and urbanization. Therefore, there is an increased demand for breeding new crops with enhanced drought tolerance and improved yields under water shortage conditions (Fulda et al., 2011; Joshi et al., 2016). The development of crops with improved drought tolerance requires knowledge of the physiological and molecular mechanisms of drought stress tolerance at different plant developmental stages (Farooq et al., 2009, 2016).

Drought stress leads to stomatal closure and reduced transpiration rates, a decline in the turgor pressure of plant cells and decreases in photosynthesis and growth. The survival of drought-stressed plants depends on the initiation and maintenance of numerous physiological and biochemical processes that are regulated by complex gene networks (Shinozaki and Yamaguchi-Shinozaki, 2007; Farooq et al., 2009). External drought stimuli are perceived by multiple membrane-based sensors, and the signals are then transmitted via various signaling pathways, causing the expression of drought-responsive genes and, ultimately, changes in the levels of drought-responsive proteins (Alvarez et al., 2014; Fang and Xiong, 2015). Functionally, drought-responsive gene products can be distinguished into proteins implicated in signaling cascades and transcriptional regulation and functional proteins that have either regulatory or metabolic roles, such as those implicated in osmolyte metabolism, scavenging and detoxification of ROS, cellular substrate proteolysis, uptake and transport of water, solutes and ions, LEA protein, and molecular chaperones (Shinozaki and Yamaguchi-Shinozaki, 2007; Fang and Xiong, 2015; Joshi et al., 2016). Currently, significant progress has been made toward understanding the complicated mechanisms of plant drought tolerance, including morphological changes, physiological responses, biochemical metabolism, and gene expression regulation. In parallel, notable progress has been made by recent applications of –omics approaches (such as genomics, transcriptomics, proteomics, and metabolomics) from different biological levels (Kissel et al., 2016). In the meantime, thousands of drought-responsive genes have been identified through Affymetrix Gene Chip technology and RNA sequencing (Hu and Xiong, 2014; Fang and Xiong, 2015). Because gene expression changes ultimately lead to protein expression changes, proteomic approaches have recently been exploited as a promising tool for the comprehensive analyses of the proteomic responses in drought-stressed plants (Hashiguchi et al., 2010). Additionally, comparative proteomics have been developed as an important approach for elucidating drought-responsive molecular mechanisms of plants with differential drought sensitivities under stress conditions (Alam et al., 2010; Alvarez et al., 2014; Hao et al., 2015; Wei et al., 2016; Cao et al., 2017). These studies will increase our understanding of drought-tolerant mechanisms in crops and pave the way for engineering water-saving and drought-tolerant crops for agricultural production.

Alfalfa (*Medicago sativa* L.) is a widely cultivated forage crop with excellent agricultural traits and significant economic value. The deep root system of alfalfa is conducive to preventing soil erosion in dry and semi-dry areas (Quan et al., 2015). Alfalfa exhibits a relatively high level of drought tolerance in comparison to other food crops (Kang et al., 2011; Tang et al., 2014). Therefore, large-scale studies intended to identify drought adaptation mechanisms in alfalfa have been investigated using physiological, genomic and agronomic approaches. Physiological responses of drought-stressed alfalfa have been studied by Wang W.B. et al. (2009), He et al. (2012), and Mouradi et al. (2016). Molecular responses of alfalfa to drought stress have been analyzed at the gene expression level by Tang et al. (2014) and Bao et al. (2016). Kang et al. (2011, 2012), and Quan et al. (2015) have reported that different drought-tolerant alfalfa varieties exhibited common and divergent responses to drought stress at the physiological, morphological, and transcriptional levels. Furthermore, proteomics has been applied to explore the molecular mechanism associated with drought tolerance of different organs of alfalfa, such as leaves (Aranjuelo et al., 2011; Ma et al., 2016) and roots (Atikur et al., 2016). Moreover, Yacoubi et al. (2011, 2013) investigated the impact of PEG imbibition on the alfalfa proteome during seed germination. Ma et al. (2017) reported the proteomic changes of germinating alfalfa seeds under PEG and NaCl stresses. Previously reported alfalfa proteomics studies were mainly using two-dimensional gel electrophoresis (2-DE) (Aranjuelo et al., 2011; Yacoubi et al., 2011; Yacoubi et al., 2013; Atikur et al., 2016; Ma et al., 2016, 2017). However, 2D-gel-based approaches had a disadvantage in the identification and accurate quantification of proteins from multiple samples. Compared with gel-based techniques, iTRAQ-based quantitative proteomics can identify more numerous proteins and quantify them more efficiently and accurately (Karp et al., 2010; Nogueira et al., 2012). To date, however, quantitative proteomics studies on drought-stressed alfalfa roots based on i TRAQ analysis have not been reported. Moreover, a comparative physiological and proteomic analysis to understand the differential responses of two contrasting alfalfa varieties to drought stress are rare.

Roots are essential organs that provide the plant with water and nutrients. The sensitivity or tolerance of a plant to unfavorable environmental conditions is highly associated with its root development modulation and the corresponding response strategies to stress (Sengupta et al., 2011). PEG, a non-absorbable, non-metabolized and non-toxic osmotic agent, is widely used to induce osmotic stress in plants by declining the water potential of nutrient solutions, making less water available to the plant roots (Joshi et al., 2011). It has been rationally well established that PEG solutions are more appropriate to simulate water deficit in plants and to assess the drought tolerance potential of plants at seedling stage (Hadi et al., 2014). In this study, comprehensive and comparative analyses of physiology and proteomic changes in the roots of two alfalfa varieties that contrast in their tolerance to drought were compared under PEG-induced osmotic stress. Our objective was to decipher differential physiological and proteomic responses of two different drought-tolerant alfalfa varieties to gradual osmotic stress. We analyzed the root activity, the accumulation of osmolytes and antioxidants, the degree of lipid peroxidation, the activities of antioxidant enzymes, and protein abundance changes in the roots of two alfalfa varieties, *Medicago sativa* L. cv. Longzhong (drought-tolerant) and *Medicago sativa* L. cv. Gannong No. 3 (drought-sensitive) at the seedling stage under osmotic stress simulated with PEG-6000. These results provide critical insights into the drought tolerance mechanisms of *Medicago sativa*.

## Materials and Methods

### Plant Growth and Treatment Conditions

Two alfalfa varieties contrasting in drought tolerance, *Medicago sativa* L. cv. Longzhong and cv. Gannong No. 3, were used for this experiment. Drought-tolerant Longzhong is a native variety that is suited for arid regions where the long-term annual precipitation is 250–400 mm (Fan et al., 2015), while drought-sensitive Gannong No. 3 is an improved variety that is suited for irrigated areas (Yang et al., 2016). Seeds of the two alfalfa varieties were provided by the Key Laboratory of Grassland Ecosystems of the Ministry of Education. The seeds were surface sterilized with 10% sodium hypochlorite solution and repeated washes with sterilized distilled water. The washed seeds were sown in a nutritional bowl (9 cm × 11 cm) filled with 450 g sand and then placed in nutritional pots (25 cm × 15 cm × 10 cm) and maintained in a growth chamber under controlled conditions (day/night cycle: 16/8 h, 25/20°C; 60 ± 5% relative humidity; and 450 μmol m^-2^ s^-1^ PPFD). After 2 weeks of growth, seedlings were transferred into full-strength Hoagland’s nutrient solution (Hoagland and Arnon, 1950). The hydroponic culture period lasted for 5 weeks, and the nutrient solution was changed once a week during this period. The solutions in all nutritional pots were bubbled with air by an air pump to provide O_2_ and then mixed to ensure homogeneous nutrient partitioning.

### Stress Treatments

The physiological responses of alfalfa seedlings were studied by transferring part of the 7-week-old alfalfa seedlings into a nutrient solution with -1.2 MPa osmotic potential, which was prepared using PEG 6000, according to the results of a prior experiment and based on the equations reported by Michel and Kaufmann (1973). The seedlings not subjected to osmotic stress were used as the control (0 MPa). The osmolality of the solution was determined by a VAPRO^®^ Vapor Pressure Deficit Osmometer (WESCOR Inc., United States). Before treatment, nutrient solutions containing PEG 6000 were placed in a water bath preset at 30°C for quick mixing. The solutions were continuously stirred with a glass rod, and after half an hour, they were transferred to each pot to a total volume of 200 mL. The nutritional pots were cleaned daily to ensure no residual nutrient solution remained before adding new nutrient solutions. At 0, 3, 6, 9, 12, and 15 days of the stress treatment, the harvested roots were immediately frozen in liquid nitrogen and stored at -80°C for measurement of other physiological parameters except for root activity. All treatments for non-stressed and stressed plants were arranged randomly inside growth chambers and replicated three times in three nutritional pots. Based on the results of the above experiment, some key physiological responses demonstrated that 9 days was an optimum treatment duration for the drought response studies of the two alfalfa varieties with contrasting tolerance. Therefore, root samples collected from plants 9 days after exposure to osmotic stress were harvested for further iTRAQ-based proteomics analysis and verification of differentially accumulated protein (DAP) genes using RT-qPCR analysis.

### Physiological Measurements

The root activity, the contents of osmolytes and antioxidants, the levels of MDA, H_2_O_2_, OH^•^, and O_2_^•-^, the activities of antioxidant enzymes and T-AOC were measured in each treatment group. The root activity was measured by the TTC method (Steponkus and Lanphear, 1967). The soluble protein content was measured using bovine serum albumin as described by Bradford (1976). The soluble sugar content was measured according to Buysse and Merckx (1993). The free proline content was determined using the acid ninhydrin method (Bates et al., 1973). The MDA content was measured with TBA reaction according to Heath and Packer (1968). The level of H_2_O_2_ was measured according to Willekens et al. (1997). The production of OH^•^ was determined as described by Liu et al. (2009) using TBA and acetic acid. The production of O_2_^•-^ was determined following the method of Elstner and Heupel (1976). AsA and DHA were measured at 525 nm according to Law et al. (1983). GSSG and GSH were assayed at 412 nm according to Griffith (1980). The levels of the above physiological parameters were all calculated based on fresh weight (FW). The enzymes were extracted from root tissues according to Chao et al. (2010). The extraction procedure of the enzymes was carried out at 4°C. The SOD activity was measured at 560 nm according to Giannopolitis and Ries (1977). The POD activity was assayed at 470 nm as described by Chance and Maehly (1955). The activities of CAT and APX were assayed by the method of Nakano and Asada (1981). The GR activity was assayed as described by Foster and Hess (1980). The T-AOC was measured as described by Liu et al. (2009). The antioxidant enzymatic activities (including SOD, POD, CAT, APX, and GR) and T-AOC were expressed as in unit mg^-1^ protein.

### Protein Extraction and Quality Testing

Total proteins were extracted from the non-stressed and stressed root tissues of two alfalfa varieties with three biological replicates (each containing 500 mg alfalfa roots) using the cold acetone method as described by Hao et al. (2015) with some modifications. Samples were ground to a powder in liquid nitrogen and lysed with 2 mL lysis buffer containing 8 M urea, 2% SDS, and 1x Protease Inhibitor Cocktail (Roche Ltd. Basel, Switzerland). Then, the solution was kept on ice for 30 min prior to centrifugation (4°C, 18,000 × *g*). After that, the supernatant was transferred into a new tube and precipitated with 10% TCA/90% acetone, followed by incubation at -20°C overnight. Pellets were washed three times with acetone. Finally, the precipitate was dissolved in 8 M urea under ultrasound irradiation. Protein was quantified using the bicinchoninic acid protein assay kit (23227, Pierce, United States), and absorbance was determined at 562 nm using a microplate reader (SpectraMax iD3, Molecular Devices, United States). Protein quality was examined with SDS-PAGE (P0012A, Beyotime, China).

### Protein Digestion and iTRAQ Labeling

The solution was transferred to a new tube and adjusted to 100 μL using 8 M urea, mixed with 11 μL 1 M DTT, and incubated at 37°C for 1 h followed by centrifugation at 4°C and 14,000 × *g* for 10 min. The supernatant was incubated in a dark room for 20 min after the addition of 120 μL 55 mM iodacetamide. After that, the supernatant was washed using 100 μL 100 mM TEAB and centrifugated at 14,000 × *g* for 10 min at 4°C followed by discarding the eluate. This washing step was repeated three times. Protein digestion was conducted using trypsin (V511C, Promega, United States) at 37°C overnight, and peptides were dried in a centrifugal vacuum concentrator. Twelve samples were labeled with iTRAQ tags (117, 118, 119, and 121) three times using the iTRAQ 8-plex labeling kit (Applied Biosystems, Foster City, CA, United States) according to the manufacturer’s instructions. More precisely, non-stressed Gannong No. 3 (GC), stressed Gannong No. 3 (GT), non-stressed Longzhong (ZC) and stressed Longzhong (ZT) were labeled with 117 tag, 118 tag, 119, tag and 121 tag, respectively.

### Strong Cation Exchange (SCX) and LC-MS/MS Analysis

Sample fractionation was performed before LC-MS/MS analysis using SCX chromatography on an Ultimate 3000 HPLC system (Thermo DINOEX, United States). The iTRAQ labeled peptide mixtures were dissolved with 100 μL Buffer A (25 mM NaH_2_PO_4_ in 25% ACN, pH 2.7). After the peptides were flowed into a 4.6 mm I.D. ×250 mm Ultremex SCX fractionation column (Phenomenex), the retained peptides were eluted at a flow rate was maintained at 1 mL/min with a gradient of Buffer A for 10 min, from 5 to 60% Buffer B (25 mM NaH_2_PO_4_, 1 M KCl in 25% ACN, pH 2.7) for 27 min, and then eluted using from 60 to 100% Buffer B for 1 min. The system was kept at 100% Buffer B for 1 min and then equilibrated with Buffer A for 10 min before the next injection. The elution was monitored by measuring absorbance at 214 nm, and the fractions were collected every minute. The eluted peptides were pooled into 12 fractions. Each fraction was desalted with a ZipTip C18 column (Waters) and then vacuum-dried. Each dried fraction was resuspended in 30 μL Buffer C (5% ACN; 0.1% FA) and centrifuged at 12000 × *g* for 10 min, the final concentration of peptide was about 0.5 μg/μL on average. A 10 μL peptide was loaded on an Eksigent nano LC System (SCIEX, USA) with a trap C18 column (5 μm, 100 μm × 2 cm). The peptides were eluted onto an analytical C18 column (3 μm, 75 μm × 15 cm). The samples were loaded at 5 μL/min for 10 min, then the 78 min gradient was run at 300 nL/min starting from 2 to 35% Buffer D (95% ACN, 0.1% FA), and the following gradient schedule was then initiated: 60% Buffer D at 5 min; 80% Buffer D at 2 min and maintained for 4 min; and finally return to 5% Buffer D in 1 min.

The fractions were analyzed by a TripleTOF 5600+ System (SCIEX, United States) fitted with a Nanospray III source and a pulled quartz tip as the emitter (New Objectives, Woburn, MA, United States). The interface temperature and ionization tip voltage were set at 150°C and 2.5 kV, respectively. Nebulizer gas and curtain gas were set to 10 and 35 psi, respectively. The MS was operated in sensitive mode for TOF MS scans. For information-dependent data acquisition (IDA), survey scans were acquired in 250 ms and as many as 30 product ion scans were collected if exceeding a threshold of 120 counts per second (cps) and with a 2+ to 5+ charge-state. Total cycle time was set to 3.3 s. The Q2 transmission window was 100 Da for 100%. Four time bins were summed for each scan at a pulser frequency value of 11 kHz by monitoring of the 40 GHz multichannel TDC detector with four-anode channel detect ion (Chen et al., 2016). A sweeping collision energy setting of 35 ± 5 eV coupled with iTRAQ adjust rolling collision energy was applied to all precursor ions for collision-induced dissociation. Precursor ions were excluded from reselection for 15 s (1/2 of average peak width).

### Database Search and Protein Quantification

All of the mass spectrometry data were obtained using Bruker Daltonics micrOTOF control and converted into MGF files using Proteome Discovery 1.2 (Thermo, Pittsburgh, PA, United States). The MGF files were analyzed against a *Medicago sativa* reference transcriptome containing 52,646 sequences (Source: NCBI [BioProject ID: PRJNA396488]) using the Mascot search engine (Matrix Science, London, United Kingdom; version 2.3.02). The search parameters were set as follows: trypsin as the cleavage enzyme; one max missed cleavages; fragment mass tolerance was set at ± 0.05 Da, and the peptide mass tolerance was set to 20 ppm; monoisotopic as the mass values; Gln → pyro-Glu (N-term Q) and Oxidation (M) as variable modifications and Carbamidomethyl (C), iTRAQ 8 plex (N-term) and iTRAQ 8 plex (K) selected as fixed modifications. Only peptides with a false discovery rate (FDR) estimation ≤ 1% and a 95% confidence interval were counted as being successfully identified. Every confident protein was identified by at least one unique peptide. All MS-based proteomic data related to this study has been public available on integrated Proteome resources (iProX)^1^ with ID IPX0000979000/ PXD008922. Proteins containing at least two unique spectra were selected for quantification of proteins (Wang et al., 2016). The relative quantification of proteins was based on the weighted average of the reporter ion intensity, which represents the relative abundance of peptides. The protein ratio (fold change) originated from the non-stressed and stressed samples through reporter ion ratios labeled with different isotopes as described above. The fold changes were calculated as the average ratios of GT to GC and ZT to ZC, and ratios for each protein in each comparison were normalized to 1 (Xie et al., 2016). Then, the differences between the groups were compared by a paired *t*-test and a *P*-value less than 0.05 was defined as significant DAPs. For quantitative changes, a 1.5-fold cut-off was set to determine proteins whose accumulation was significantly increased or decreased. GO analysis^2^ was used for functional annotation of DAPs identified to describe the biological processes, cellular component, and molecular function involved in the response to osmotic stress. DAPs in biological pathways and signal transduction pathways were allocated using KEGG pathway analysis^3^. Significant pathway enrichment was performed using a hypergeometric test, and a *P*-value less than 0.05 was defined as statistically significant.

### RNA Extraction, cDNA Synthesis, and RT-qPCR Analysis

Total RNA was extracted from non-stressed and stressed root tissues of two alfalfa varieties with three biological replicates (each containing 100 mg alfalfa roots) using the RNA simple Total RNA Kit (TIANGEN BIOTECH, Co., Ltd, Beijing, China) based on the kit instructions. cDNA was reverse transcribed from 1 μg of total RNA using a First Strand cDNA Synthesis Kit (TaKaRa, Dalian, China) (Niu et al., 2017). Specific RT-qPCR primers were designed using Primer 3^4^ (**Table 1**). RT-qPCR was conducted with an iQ^TM^ 5 real-time PCR machine (Bio-Rad, United States) using the SYBR^®^ Premix Dimer Eraser^TM^ (TaKaRa, Dalian, China). Each 20 μL reaction mixture contained 2 μL template cDNA, 10 μL of 2× SYBR^®^ Premix Dimer Eraser, 0.8 μL of each primer (10 μM), 6.0 μL dd H_2_O, and 0.4 μL of ROX reference Dye. The amplification reaction conditions were as follows: 95°C for 3 min followed by 40 cycles of 95°C for 10 s and 60°C for 30 s (Niu et al., 2017). The levels of relative gene expression were calculated by the ddCt algorithm using the alfalfa *MtGAPDH* gene as the housekeeping gene (Zhang et al., 2003). Three biological replicates were performed for each sample.

**Table 1:** Sequence of primers used in RT-qPCR.

Name of gene	Primer sequences (5′-3′) (forward/reverse)	Amplication product length (bp)	Amplication product Tm (°C)
NAD-dependent aldehyde dehydrogenase (ALDH) family protein (*MtALDH*)	GAGTTGTGGAGGGTGCAGTT/AGCTCCAGAACGACTGGTGT	142	84.37
6-Phosphofructokinase (*MtPFK*)	GCAATCAGTGCTGCTCATGT/GGCTGCTGAGTGTTGAATGA	112	85.71
Glutathione S-transferase (*MtGST*)	ATGAGAAAGAGCGCGAGAAG/TTGTCAGCAGGGAACAACTG	175	83.61
Aldose reductase (*MtAR*)	CTCCTTTAGGCTCACCAGGA/TCCTGCTTGTAGACCCCATC	125	82.03
Chitinase (*Mtchitinase*)	GCCGGTAGAGCCATTAATCA/ATATCCGGGTACCCTCCTTG	192	82.65
MLP-like protein (*MtMLP*)	CAAAACACCCGCTGATAGGT/GTGTGATGCCAATCTTCACC	116	83.0
Pirin-like plant protein (*MtPLPP*)	TCACCATGATGCCAGGAAC/GTGAGCCACAATTGGTGATG	186	80.57
Lysine-ketoglutarate reductase/saccharopine dehydrogenase (*MtLKR*)	GCTGTGGTGCACAGGAGATA/CTTTGGGCTCAACCATGTCT	168	82.22
Expansin-B1-like protein (*Mt EBLP*)	GCAATGTTGCTGGTGTGTCT/CCACTACGGTTGCTCCATTT	114	83.51
Glucan endo-1,3-beta-glucosidase (*MtGI*)	GTACCCGCCGTCTAAAGGTT/GAACCATCCCAAACCACAAC	195	79.44
Hydroxymethylglutaryl-CoA synthase (*MtHMCS*)	GGACTTGTCGTCTGCACTGA/GCCATATGACTTCCCCTCAA	140	86.56
Glucosyltransferase (*MtGTF*)	GCTTCAAAGGGTCACACTGTC/ACCTGGTGGGAGTTGCTCTA	138	82.46
Glyceraldehyde-3-phosphate dehydrogenase (*MtGAPDH*)	GGCTGTAGGCAAAGTGCTTC/CTTGATGGCAGCCTTGATCT	145	84.64


### Statistical Analysis

All physiological and RT-qPCR data analyses were performed using the SPSS Statistical Software (version 19.0; SPSS Institute Ltd, United States), and the significance of differences were tested using Fisher’s protected least significant difference test (LSD) with a *P*-value ≤ 0.05 set as statistically significant.

## Results

### Effect of Osmotic Stress on Root Activity and Osmolyte Contents

**FIGURE 1:**
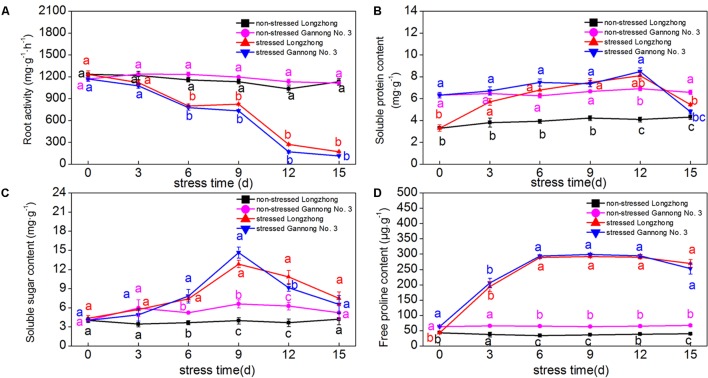
Changes of root activity **(A)** and the contents of soluble protein, soluble sugar, and proline **(B–D)** in two alfalfa varieties under non-stressed and stressed conditions at different time points. Different letters above line graphs indicate significant difference among treatments at a given day of treatment (*P* ≤ 0.05), and vertical bars indicate ± SE of mean (*n* = 3).

Osmotic stress resulted in a significant decrease in root activity, but a significant increase in proline content. The soluble protein and soluble sugar contents of both varieties showed a trend of first increasing and then decreasing with the stress time increasing (**Figures 1A–D**). Overall, the osmolyte contents of Gannong No. 3 were significantly greater than those of Longzhong under the control conditions; but there was no significant change between varieties under stress conditions. The soluble sugar and proline contents peaked at 9 days under stress conditions, while the soluble protein content peaked at 12 days. Longzhong showed a greater increase in osmolyte accumulation than Gannong No. 3 during the osmotic stress period.

### Effect of Osmotic Stress on Levels of Lipid Peroxidation

**FIGURE 2:**
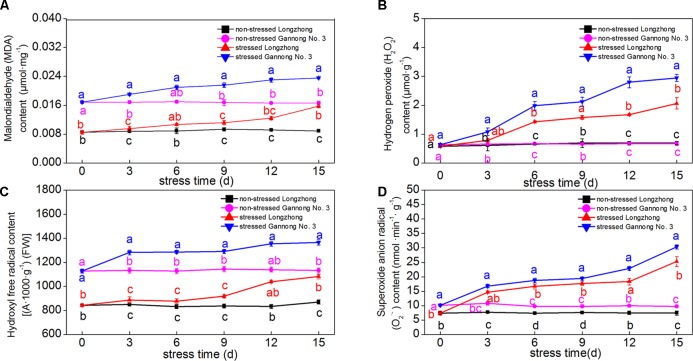
Changes of the levels of MDA, H_2_O_2_, OH^•^ and O_2_^•-^ **(A–D)** in two alfalfa varieties under non-stressed and stressed conditions at different time points. Different letters above line graphs indicate significant difference among treatments at a given day of treatment (*P* ≤ 0.05), and vertical bars indicate ± SE of mean (*n* = 3).

The contents of MDA, H_2_O_2_, OH^•^ and O_2_^•-^ of both varieties presented an increasing trend with increasing exposure to osmotic stress. Gannong No. 3 exhibited significantly higher levels of MDA, H_2_O_2_, OH^•^ and O_2_^•-^ than those of Longzhong (**Figures 2A–D**). Gannong No. 3 showed a greater increase in the MDA contents than Longzhong during osmotic treatments. After 6 and 9 days of osmotic stress, the increases in H_2_O_2_ and OH^•^ contents were greater in Gannong No. 3 than those in Longzhong. After 15 days of osmotic stress, the levels of MDA, H_2_O_2_, OH^•^ and O_2_^•-^ in Gannong No. 3 were all significantly higher than those of Longzhong by 49.50, 42.66, 26.19, and 20.09%, respectively (*P* < 0.05).

### Effect of Osmotic Stress on the Activities of Antioxidant Enzymes and T-AOC

**FIGURE 3:**
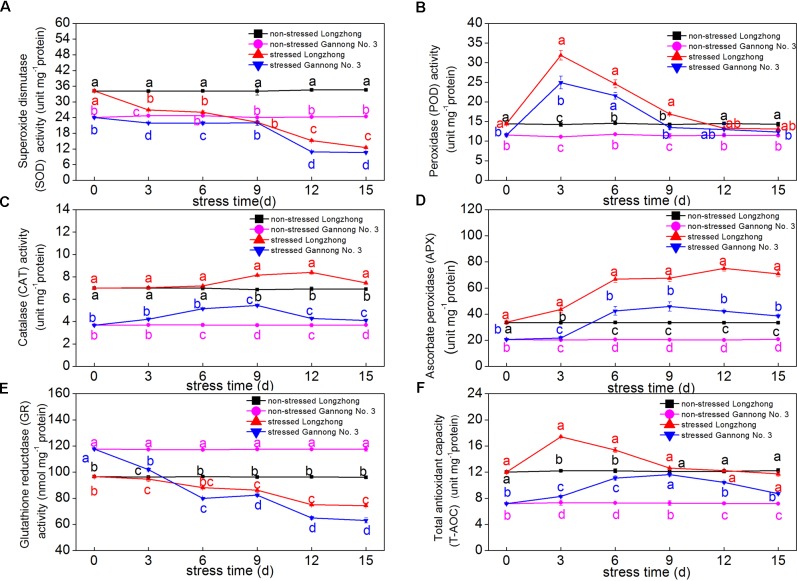
Changes of the activities of antioxidant enzymes (SOD, POD, CAT, APX, and GR) **(A–E)** and total antioxidant capacity (T-AOC) **(F)** in two alfalfa varieties under non-stressed and stressed conditions at different time points. Different letters above line graphs indicate significant difference among treatments at a given day of treatment (*P* ≤ 0.05), and vertical bars indicate ± SE of mean (*n* = 3).

With the extension of stress time, the SOD and GR activities in both varieties declined continuously, while the levels of POD, CAT, APX and T-AOC first increased and then decreased. Longzhong exhibited significantly higher activities of antioxidant enzymes (SOD, CAT, and APX) and T-AOC than those of Gannong No.3 under both control and stressed conditions (**Figures 3A–F**). The SOD and GR activities in both varieties sharply decreased after 9 days of osmotic stress (**Figures 3A,E**). After 15 days of PEG treatment, the GR activities of Longzhong and Gannong No. 3 dramatically decreased by 22.60 and 46.33%, respectively, compared with control plants. The POD activities of Longzhong and Gannong No. 3 peaked at 3 days after stress, with 124.18 and 124.12% higher than that of control plants, respectively. The T-AOCs of Longzhong and Gannong No. 3 peaked at 3 and 9 days after treatment, respectively. The increase amplitudes of POD activities and T-AOCs in both varieties all declined when the stress duration exceeded 9 days (**Figures 3B,F**). The CAT and APX activities of Longzhong peaked at 12 days after treatment, while those of Gannong No. 3 all peaked at 9 days after stress (**Figures 3C,D**). After 15 days of treatment, the levels of SOD, CAT, APX, GR, and T-AOC in Longzhong were all significantly greater than those of Gannong No. 3 by 17.94, 81.61, 84.28, 18.00, and 34.64%, respectively (*P* < 0.05).

### Effect of Osmotic Stress on the Contents of Antioxidants

**FIGURE 4:**
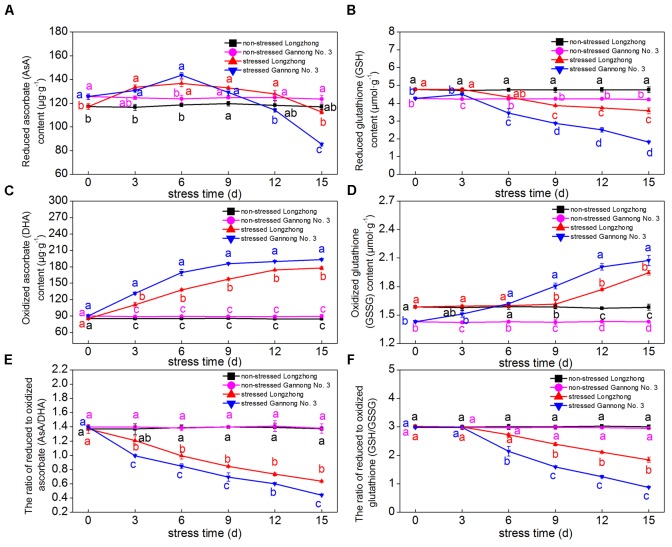
Changes of the contents of antioxidants (AsA, DHA, GSH, GSSG) **(A–D)**, and the ratios of AsA/DHA **(E)** and GSH/GSSG **(F)** in two alfalfa varieties under non-stressed and stressed conditions at different time points. Different letters above line graphs indicate significant difference among treatments at a given day of treatment (*P* ≤ 0.05), and vertical bars indicate ± SE of mean (*n* = 3).

With the increase in treatment days, the AsA and GSH contents of both varieties showed a trend of decline, while the DHA and GSSG contents showed a trend of gradual increase. The ratios of AsA/DHA and GSH/GSSG significantly decreased under osmotic stress (**Figures 4A–F**). After 3 days of osmotic stress, the AsA contents of both varieties increased slightly, but the DHA contents increased significantly, resulting in a significant decrease in the AsA/DHA ratio; however, the AsA/DHA ratio of Longzhong was significantly higher than that of Gannong No. 3 during the treatments (**Figures 4A,C,E**). After 6 days of osmotic stress, the GSH contents and the GSH/GSSG ratio of both varieties decreased significantly. After 9–15 days of osmotic stress, Longzhong showed higher GSH contents and GSH/GSSG ratio than Gannong No. 3 (**Figures 4B,F**).

### Primary Data Analysis and Protein Detection

Given the physiological traits of alfalfa seedlings under osmotic stress, root proteomic changes in the seedlings of two alfalfa varieties exposed to -1.2 MPa PEG 6000 for 9 days were analyzed using iTRAQ-LC/MS-MS. A total of 225,188 spectra were generated through the iTRAQ analysis. Among these spectra, we detected a total of 52,774 spectra corresponding to known spectra, 38,474 spectra corresponding to unique spectra, 16,755 peptides, 13,916 unique peptides, and 4272 proteins (Supplementary Figure 1). The distribution of the number of peptides defining each protein is shown in Supplementary Figure 2, and over 65.5% of the proteins included at least two peptides.

### Identification and Functional Classification of DAPs

A total of 1901 proteins were identified and quantified in roots of two alfalfa varieties at a 95% confidence level in three biological replicates (Supplementary Data Sheets 1–4). GO annotations indicated that all 1901 identified proteins were involved in a wide range of biological processes, cellular components, and molecular functions. The 1901 identified proteins were annotated for 20 biological processes. Among these categories, metabolic process, cellular process, single-organism process, response to stimulus and localization were the most common biological processes. The results of the cellular component annotation indicated that 48.11% of the proteins were located in cells and cell parts, 16.42% in organelles, 9.16% in organelle parts, and 8.78% in membranes. Moreover, for annotations of molecular functions, 48.13% of the proteins were associated with catalytic activity and 41.16% with binding (Supplementary Figure 3).

**FIGURE 5:**
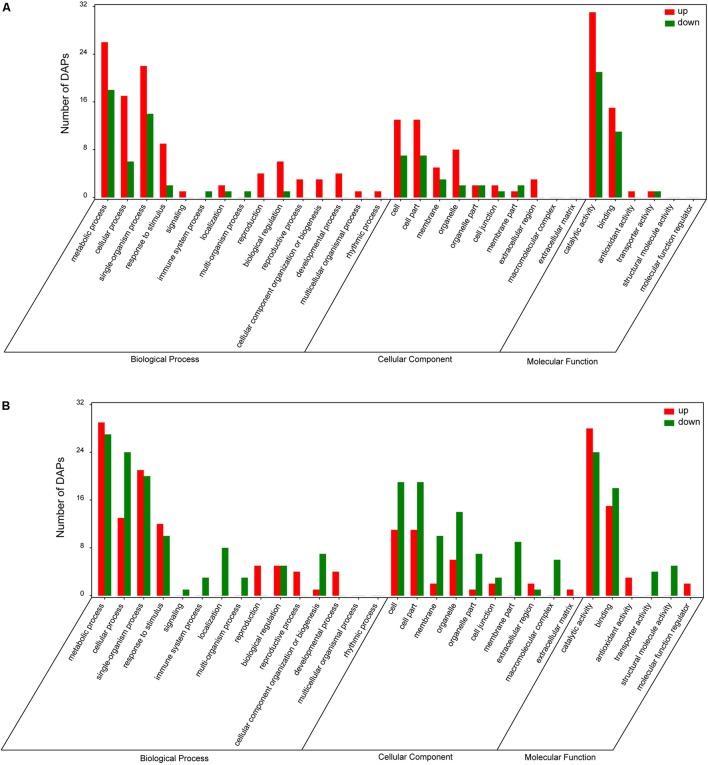
Gene Ontology (GO) analysis of DAPs in roots of *Medicago sativa* L. cv. Longzhong **(A)** and cv. Gannong No. 3 **(B)** under PEG-induced osmotic stress (red and green bars represent up- and down-regulated proteins, respectively).

**FIGURE 6:**
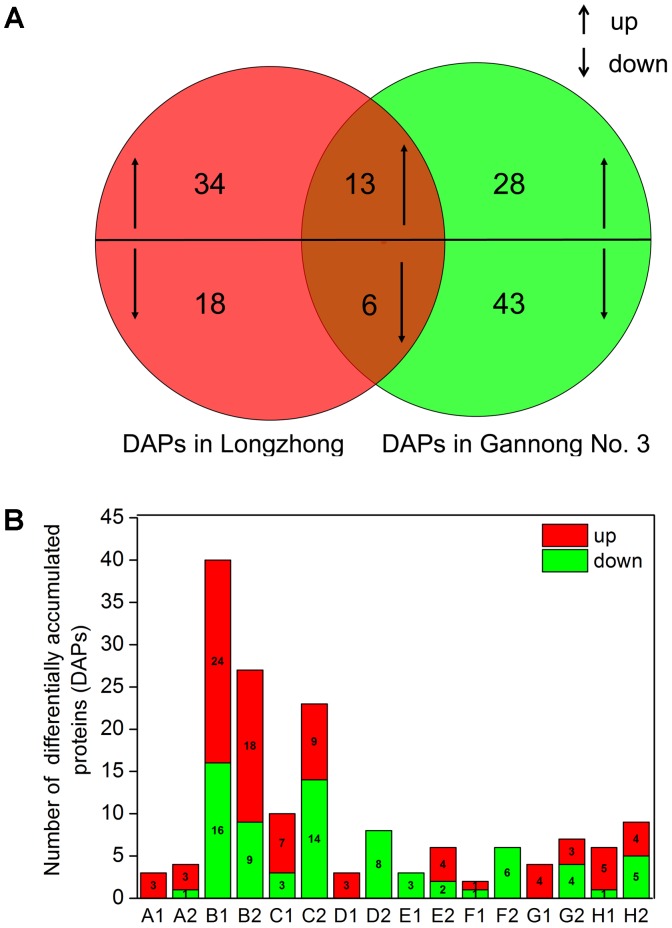
Graphical representation and functional cataloging of DAPs **(A)** Venn diagram shows the number of common, significantly up-regulated and down-regulated proteins in two alfalfa varieties under PEG-induced osmotic stress (up- and downward arrows represent up-regulated and down-regulated proteins, respectively). **(B)** The column chart shows the functional categories of the DAPs including carbohydrate and energy metabolism (A1, A2), stress and defense (B1, B2), protein metabolism (C1, C2), membrane and transport (D1, D2), signal introductions (E1, E2), transcription (F1, F2), cell wall and cytoskeleton metabolism (G1, G2), in addition to and unknown functions (H1, H2) (red and green bars represent up- and down-regulated proteins, respectively. A1, B1, C1, D1, E1, F1, G1 and H1 represent correspondingly functional categories of the DAPs in *Medicago sativa* L. cv. Longzhong; and A2, B2, C2, D2, E2, F2, G2, and H2 represent correspondingly functional categories of the DAPs in *Medicago sativa* L. cv. Gannong No. 3).

To identify DAPs, the results of iTRAQ analysis were divided into two different ratios including ZT/ZC (stressed Longzhong compared to control) and GT/GC (stressed Gannong No. 3 compared to control). The ZT/ZC and GT/GC ratios indicated the effects of PEG-induced osmotic stress on the Longzhong and Gannong No. 3, respectively. A total of 142 DAPs were identified from two alfalfa varieties. Among these proteins, 19 proteins (13 up-regulated and 6 down-regulated) were shared by both varieties, 52 proteins (34 up-regulated and 18 down-regulated) only presented in Longzhong, and 71 proteins (28 up-regulated and 43 down-regulated) only presented in Gannong No. 3 (Supplementary Data Sheets 5, 6). The GO annotation and enrichment analysis indicated that all identified DAPs could be related to 15 biological processes, 10 cellular components and 6 molecular functions. The number of down-regulated proteins implicated in the prevalent biological processes (including metabolic process, cellular process, single-organism process, and response to stimulus), and also located in main cellular components (including cell, cell part, membrane, organelle, organelle part) were higher in Gannong No.3, compared with those in Longzhong (**Figures 5A,B**). Venn diagram analysis was illustrated the numbers of common, significantly up-regulated and down-regulated proteins in both varieties (**Figure 6A**). These results indicated that significant differences exist in the response of the two alfalfa varieties to osmotic stress. The 142 DAPs were classified into the following eight groups according to their biochemical functions: energy and carbohydrate metabolism (7 DAPs, 4.93%), stress and defense (57 DAPs except for 10 DAPs shared by two varieties, 40.14%), protein metabolism (29 DAPs except for 4 DAPs shared by two varieties, 20.42%), membrane and transport (11 DAPs, 7.75%), signal transduction (9 DAPs, 6.34%), transcription (8 DAPs, 5.63%), cell wall and cytoskeleton metabolism (9 DAPs except for 2 DAPs shared by two varieties, 6.34%), and unknown functions (12 DAPs except for 3 DAPs shared by two varieties, 8.45%) (**Figure 6B**). There was a large decrease in protein processing and ribosome biogenesis, membrane and transport, transcription, as well as cell wall and cytoskeleton metabolism in Gannong No. 3 variety.

### Validation of Select DEP Expression Changes by RT-qPCR

**FIGURE 7:**
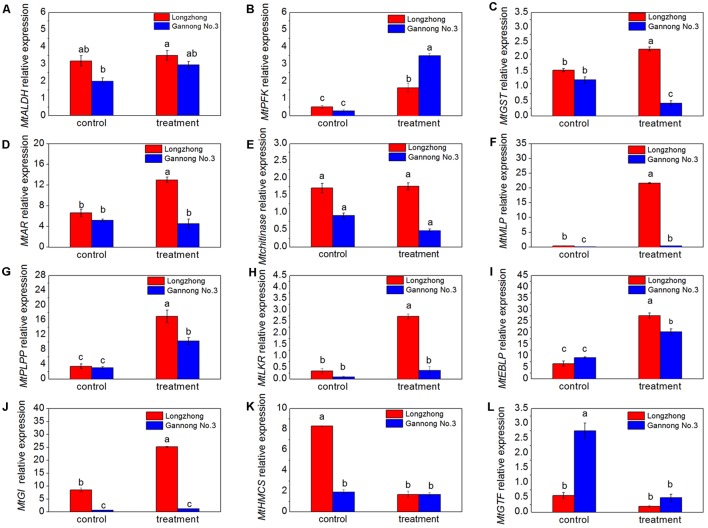
Transcriptional expression of twelve DAPs encoding genes in roots of two alfalfa varieties. **(A–L)** Changes in expression of *MtALDH*, *MtPFK*, *MtGST*, *MtAR*, *Mtchitinase*, *MtMLP*, *MtPLPP*, *MtLKR*, *MtEBLP, MtGI*, *MtHMCS*, and *MtGTF* in roots of two alfalfa varieties after 9 days of PEG-induced osmotic stress. Different letters above columns indicate significant difference (*P* ≤ 0.05), and vertical bars above columns indicate ± SE of mean (*n* = 3).

The expression of twelve genes, related to carbohydrate metabolism (*MtALDH* and *MtPFK*), stress and defense (*MtGST*, *MtAR*, *Mtchitinase*, *MtMLP*, *MtPLPP*, *MtGI*, and *MtGTF*), protein metabolism (*MtLKR*), and cell wall and cell cytoskeleton metabolism (*MtEBLP* and *MtHMCS*), were determined by RT-qPCR to confirm the correlation between the expression changes of RNA and corresponding proteins (**Figures 7A–L**). Compared to the control, the expression levels of nine genes (*MtALDH*, *MtPFK*, *MtMLP*, *MtPLPP*, *MtLKR*, *MtEBLP, MtGI*) were increased while the expression levels of *MtHMCS* and *MtGTF* were decreased under osmotic stress conditions (**Figures 7A,B,F–L**). There was no significant change in the expression level of *Mtchitinase* gene (**Figure 7E**). Moreover, osmotic stress significantly induced expression of *MtPFK, MtMLP, MtPLPP*, and *MtEBLP* in both Longzhong and Gannong No. 3 (**Figures 7B,F,G,I**). The expressions of *MtGST*, *MtAR*, *MtLKR*, and *MtGI* were significantly induced by osmotic stress in Longzhong (**Figures 7C,D,H,J**). However, the expressions of *MtGST* and *MtGTF* in Gannong No. 3 and *MtHMCS* in Longzhong were suppressed by osmotic stress (**Figures 7C,L,K**).

## Discussion

Drought is a critical environmental factor in limiting plant growth and agricultural productivity (Quan et al., 2015). Alfalfa (*Medicago sativa* L.) is widely grown on non-irrigated drylands and rangelands due to its deep root system (Anower et al., 2015). Therefore, alfalfa is a promising candidate to understand the physiological and molecular responses of plant roots to drought stress (Kang et al., 2011; Quan et al., 2015). To elucidate the differential responses of two alfalfa varieties with contrasting drought tolerance, comparative physiological and proteomic analyses were performed in our research.

### Osmotic Stress Affects Physiological Changes in the Roots of Alfalfa Seedlings

Plant root systems have evolved various defense mechanisms against drought stress at morpho-physiological and molecular levels, such as root architecture adaptation, osmotic adjustment, enhancement of the antioxidant defense systems, maintenance of cell membrane stability, and the expression of drought-responsive genes and proteins (Fang and Xiong, 2015; Khoyerdi et al., 2016; Cao et al., 2017). In this study, we exposed alfalfa plants to osmotic stress for different treatment durations in hydroponic solutions containing PEG. Our data showed that after 3–12 days of osmotic stress, the accumulation of osmolytes (soluble protein, soluble sugar and proline) in both varieties were significantly increased; however, there were no marked differences in the osmolyte contents between them. Moreover, compared with Gannong No. 3, Longzhong exhibited lower levels of osmolyte contents under control conditions but increased accumulation of osmolytes under stressed conditions. These results revealed that Longzhong manifested higher osmotic adjustment capacity than Gannong No. 3 in response to osmotic stress.

As a product of lipid peroxidation, MDA can reflect the degree of membrane lipid peroxidation (Upadhyaya et al., 2007). ROS, such as H_2_O_2_, OH^•^, and O_2_^•-^, can destroy normal metabolism through oxidative damage to lipids, proteins and DNA (Farooq et al., 2009). Previous studies reported that the levels of MDA, H_2_O_2_, OH^•^ and O_2_^•-^ significantly increased in response to drought stress (Upadhyaya et al., 2007; Farooq et al., 2016; Cao et al., 2017). In this study, the MDA and H_2_O_2_ contents of Gannong No. 3 were significantly increased after 3 days of stress, while those of Longzhong exhibited a significant increase after 6 days of stress. Furthermore, the levels of MDA, H_2_O_2_, OH^•^ and O_2_^•-^ of Gannong No. 3 were significantly higher than those of Longzhong during the experiment. All these changes indicated that Gannong No. 3 experienced more serious membrane damage under osmotic stress. Similarly, Boldaji et al. (2012) and Quan et al. (2015) found lower MDA and ROS levels in drought-tolerant alfalfa varieties. The increased ROS products can be alleviated by a concerted action of enzymatic and non-enzymatic antioxidant defense systems. These systems include *a*-tocopherol, *b*-carotene, glutathione, ascorbate, and antioxidant enzymes such as SOD, POD, CAT, APX, and GR (Halliwell, 1987). The balance of SOD and APX or CAT activities in cells is critical for determining the steady-state levels of O_2_^•-^ and H_2_O_2_ (Mittler, 2002). In accordance with the findings of Jiang and Huang (2001) and Cao et al. (2017), the activities of APX, CAT, and POD were all enhanced after 3–9 days of osmotic stress. When the stress duration exceeded 12 days, the APX and CAT activities of both varieties began to decline but were still significantly greater than those of the control, while the SOD and GR activities of the two varieties sharply began to decrease after 9 days of stress. After 15 days of severe osmotic stress, Longzhong exhibited higher enzymatic activities of SOD, CAT, APX, and GR and levels of T-AOC as compared with Gannong No. 3. These results suggested that alfalfa increases the activities of these antioxidant enzymes to maintain a balance of the generation and elimination of ROS at certain times of osmotic stress.

Additionally, a co-regulated antioxidant system could help the enhancement of antioxidant capacity. The ascorbate-glutathione (AsA-GSH) cycle is an effective antioxidant system for the detoxification of excess ROS by maintaining the ratios of AsA/DHA and GSH/GSSG. These ratios are maintained by APX, MDAR, DHAR, and GR (Asada, 1999). Jiang and Huang (2001) reported that GR activities were enhanced in tall fescue and kentucky bluegrass under drought stress. In the present study, the GR activity of Gannong No. 3 significantly decreased with extended periods of osmotic stress, while that of Longzhong began to decrease when the duration under stress exceeded 9 days. This result indicated that an earlier dynamic change in the AsA-GSH cycle of Gannong No. 3 could be induced by osmotic stress due to a drastic decrease in the GR activity. Our data also showed that when duration under stress exceeded 6 days, the ratios of AsA/DHA and GSH/GSSG were all significantly lower than those in the control, suggesting that osmotic stress negatively affects the balance of reduced to oxidized forms of ascorbic acid and glutathione in the AsA-GSH cycle. It is generally accepted that a higher reduced per oxidized ratio of ascorbic acid and glutathione is indispensable for the proper ROS scavenging in plant cells (Asada, 1999; Wei et al., 2015). As expected, drought-tolerant Longzhong showed higher ratios of AsA/DHA and GSH/GSSG than Gannong No. 3 during the treatments. Overall, the changes in the above physiological parameters indicated that 9 days of osmotic stress may be the optimal duration to study the differential response of two alfalfa varieties against stress. Moreover, the higher activities of antioxidant enzymes and ratios of AsA/DHA and GSH/GSSG in Longzhong may contribute to the protection of membrane stability from osmotic stress-induced oxidative damage. Consistent with our findings, Wang W.B. et al. (2009), Boldaji et al. (2012), and Quan et al. (2015) have reported that drought-tolerant alfalfa can inhibit the accumulation of ROS due to a stronger ROS scavenging capability. The main difference in the physiological responses of the two alfalfa varieties contrasting in drought tolerance may be the equilibrium between ROS generation and scavenging in roots. However, the synergistic mechanism of different antioxidant defense systems in different drought-tolerant alfalfa varieties remains to be further studied.

### Effect of Osmotic Stress on Protein Profiling Changes

To understand the molecular response mechanisms of alfalfa under osmotic stress, proteomes of the two contrasting alfalfa varieties were investigated using an iTRAQ-based quantitative proteomic approach. In our experiment, 142 DAPs were identified from stressed Longzhong and Gannong No. 3 roots. Among these proteins, 52 proteins were present only in Longzhong, 71 proteins were present only in Gannong No. 3, and 19 proteins were commonly present in both varieties. The functions and pathways of these drought stress-responsive (mainly LZRPs) proteins are discussed in the following section.

### Carbohydrate and Energy Metabolism-Related Proteins

Carbon is indispensable for energy circulation and survival and is continuously metabolized in plants, even under adverse conditions (Hao et al., 2015). Our data found that osmotic stress altered the abundance of three LZRPs and four GN3RPs related to energy and carbon metabolism. Of these, the increased accumulation of 6-phosphofructokinase (gi|358347084) and NAD-dependent aldehyde dehydrogenase (ALDH) family protein (gi|657394832), involved in the glycolysis and gluconeogenesis pathway, could contribute to enhance drought-tolerance of alfalfa, possibly through promoting carbohydrate metabolism and aldehyde detoxification (Končitíková et al., 2015; Yao and Wu, 2016). Lee et al. (2016) have proposed that genes implicated in fructose and mannose metabolism were up-regulated by salt stress in a rice mutant line. Consistent with this finding, we observed the increased abundance of L-idonate 5-dehydrogenase (gi|358345353), involved in fructose and mannose metabolism, indicating that the induction of this protein is associated with the drought tolerance in Longzhong under PEG stress. Sucrose synthase (gi|657380149) was found to be up-regulated in Longzhong, suggesting that starch and sucrose metabolism may make an important contribution to the response against water deficit. This finding is similar to the observation of Chen et al. (2016) in salt-stressed cotton roots. Moreover, the increased accumulation of raffinose synthase or seed inhibition protein and UDP-glucose 4-epimerase related to galactose metabolism in Gannong No.3, could suggest that galactose metabolism participate in the stress response of Gannong No.3. Taken together, the above results indicated that the enhancement of the carbohydrate metabolism could be contributed to increase energy production in the cell, which enables the roots of stressed alfalfa to normal growth under osmotic stress. However, the adaptive mechanism that maintains energy homeostasis between plant growth and defense may vary with the alfalfa varieties.

### Stress- and Defense-Related Proteins

Plants have developed an array of complex detoxification and defense mechanisms to protect cells from the oxidative damage of excessive ROS (Farooq et al., 2009; Kang and Udvardi, 2012; Ziogas et al., 2013; Fang and Xiong, 2015). Here, we observed that osmotic stress altered the abundance of 57 DAPs consisting of 17 up- and 13 down-regulated LZRPs, 11 up- and 6 down-regulated GNRPs, and 10 proteins (7 up-regulated and 3 down-regulated) shared by two varieties. Among these proteins, we found that enhanced accumulation of GST and GSH-Px in Longzhong roots, suggesting that the activation of enzymatic antioxidant systems is crucial protective mechanisms for osmotic-stressed Longzhong to reduce or scavenge ROS. Notably, we found the up-regulation of four GSTs in Longzhong but one up-regulated GST in Gannong No. 3. This result implied that GSTs may increase drought tolerance by playing a role in cellular detoxification and protecting cells from oxygen toxicity, which is more prominent in Longzhong than in Gannong No. 3. Previous studies have found that the abundance of GST was up-regulated in Al-treated roots of two *Citrus* species (Jiang et al., 2015) and drought-stressed leaves of tobacco (Xie et al., 2016). Reticuline oxidase-like protein is an enzyme involved in the formation of some secondary metabolites; meanwhile, FAD-binding berberine family protein is an oxidoreductase in the endomembrane system. Both of them play a key role in the plant’s response to stress (Belchínavarro et al., 2013). As expected, we identified increased accumulation of these two proteins in both alfalfa varieties. Aldo/keto reductase (AKR) plays a vital role in various plant metabolic reactions including detoxification of reactive aldehydes, secondary metabolism, osmolyte biosynthesis, and membrane transport. In this study, the higher abundance of three AKRs in both varieties suggested that AKRs had multiple overlapping functions in stress defense and detoxification, which agreed with the findings of Sengupta et al. (2015) and Wang et al. (2016). Overall, it can be inferred that Longzhong would more effectively activate antioxidant defense mechanisms to maintain the intracellular redox homeostasis than Gannong No. 3 under osmotic stress. In addition, pirin-like protein has been reported to possess quercetinase activity, but its biological function still remains unraveled (Widiatningrum et al., 2015). Hennessy et al. (2017) have reported that the enhanced expression of a pirin-like protein encoding gene may active the defense responses of biocontrol bacteria to the phytopathogens. In this study, the higher accumulation of pirin-like plant protein (gi|657376365) was also found in both varieties, suggesting that the protein may also be induced by osmotic stress. The increased abundance of glucan endo-1,3-beta-glucosidases (gi|657388755) in both varieties indicated that it may play a vital role in plant defense against water deficit. This result is consistent with the findings of Li et al. (2010) and Wu et al. (2015). UDP-glucuronosyltransferases (UGT) are essential for detoxification metabolism and glucuronidation process through enhancing the conversion of many lipophilic compounds to more water-soluble compounds (Rowland et al., 2013). Here, the declined accumulation of UDP-glucuronosyltransferase 2B5 (gi|357470367) in both alfalfa varieties indicated that the process of glucuronidation would be inhibited and also many toxins and endogenous substances would not be eliminated.

Metabolic adaptation of plants exposed to different stress requires sophisticated metabolic reorganization of multiple metabolic pathways. Several cytoplasmic enzymes involved in defensive secondary metabolism in roots were found to be altered under various abiotic stress conditions (Alvarez et al., 2014; Bai et al., 2016). In the current study, four proteins related to phenylpropanoid biosynthesis were induced by osmotic stress. Of these, cinnamyl alcohol dehydrogenase (CAD) (gi|399168) and peroxidase 2 (gi|971564) catalyze the key steps in phenylpropanoid biosynthesis (Chen et al., 2016). *S*-adenosyl-L-methionine: caffeic acid 3-*o*-methyltransferase (COMT) (gi|261889456) and caffeoyl-CoA 3-*O*-methyltransferase (CCOMT) (gi|30580341) are key enzymes implicated in the lignin synthesis (Gowri et al., 1991; Tu et al., 2010). These results demonstrated that the induction of the phenylpropanoid pathway is essential to the survival of plants under stress conditions (Ferrer et al., 2008). Consistent with this observation, the increased abundance of CCOMT in roots of drought-stressed and salt-stressed soybean has been reported by previous proteomics studies (Alam et al., 2010; Wei et al., 2016). However, the down-regulation of six proteins related to isoflavonoid and flavonoid metabolism, including three LZRPs (i.e., gi|75293247, gi|358348252, and gi|537296) and three GN3RPs (gi|1708426), could indicate that decreases in the activities of these enzymes may interrupt the synthesis of certain defense substances. Osmotic stress induced different abiotic stress-responsive proteins in the two contrasting alfalfa varieties. Chitinases are implicated in plant growth, development and stress responses to adverse environmental conditions. The catalytic domain of chitinases is grouped into glycosyl hydrolase family 18 (GH18) and glycosyl hydrolase family 19 (GH19), and the latter consists of the Class I, Class II, and Class IV chitinases (Wang J. et al., 2009; Xiong et al., 2010; Grover, 2012). In this study, the increased accumulation of four chitinases, including chitinase (gi|298364452) in both alfalfa varieties, GH18 protein (gi|657395923) in Longzhong, and Class I chitinase (gi|1800141) in Gannong No.3, suggested that chitinases could significantly be induced by PEG stress and play an essential role in the response of alfalfa to water deficit. These results are in agreement with the previous reports of Wang J. et al. (2009) in dehydrated tall fescue and Xiong et al. (2010) in PEG-treated rice. Previous studies have reported that the expression of LEA proteins has been correlated with enhanced plant tolerance to abiotic stress (Ochoa-Alfaro et al., 2012). In our research, the higher abundance of three LEA-related proteins (i.e., gi|657386375, gi|289540897, and gi|187710977) was only observed in Longzhong roots, suggesting that these proteins may play a role in protecting biological macromolecules and preventing excessive dehydration of roots (Shinozaki and Yamaguchi-Shinozaki, 2007; Fang and Xiong, 2015). Furthermore, the up-regulations of class-10 pathogenesis-related protein 1 (PR10-1) and two disease resistance response (DRR) proteins were only found in Gannong No. 3 roots, indicating that these proteins may participate in the defense response of Gannong No. 3 to cellular dehydration. Major latex protein (MLP)-like protein, as a positive regulator of downstream signaling, mainly responds to defense or abiotic stimulus. Ma et al. (2017) reported that the abundance of MLP-like protein in germinating alfalfa seeds declined remarkably under salt stress but almost completely unchanged under osmotic stress. However, our data found that the abundance of MLP-like protein (gi|357515827) was increased in Longzhong but was decreased in Gannong No. 3. This result indicated that MLP-like protein might participate in regulating drought stress responses but might be differentially accumulated in two alfalfa varieties with contrasting drought tolerance. Overall, the present observations imply that these induced proteins might play an important role in the activation of enzymatic antioxidant system, ROS detoxification, secondary metabolism and the induction of the most prominent stress adaptive mechanism, thus enhancing the drought tolerance of alfalfa.

### Protein Metabolism-Related Proteins

Protein turnover is a ubiquitous form of regulation to cope with unfavorable conditions (Reinbothe et al., 2010). Here, we observed 29 proteins, including 6 LZRPs (5 up-regulated and one down-regulated), 19 GN3RPs (7 up-regulated and 12 down-regulated), and 4 proteins (2 up-regulated and 2 down-regulated) shared by both varieties, were differentially accumulated in response to osmotic stress. Phosphatidylethanolamine-binding proteins (PEBPs) is implicated in the modulating of various developmental processes in plants such as seed germination and morphological transformation between shoot growth and flower structures (Wang et al., 2015). Here, the enhanced accumulation of PEBP (gi|657386640) in both varieties could indicate that it plays critical role in alfalfa adaptation to osmotic stress. However, the abundance of six proteins involved in protein processing in endoplasmic reticulum and four proteins involved in ribosome biogenesis was all decreased in roots of Gannong No. 3 exposed to PEG treatment. Specific proteolysis begins with the labeling of target proteins. Ubiquitin functions as a covalent cofactor in energy-dependent intracellular proteolysis. Ubiquitin carrier enzyme is involved in the process of protein degradation. Here, the down-regulation of five ubiquitin carrier proteins (i.e., gi|357512497, gi|357473799, gi|357473801, gi|357491529, and gi|357460519) in Gannong No. 3 suggested that the key regulatory proteins and enzymes involved in the ubiquitin proteasome pathway would be selectively degraded, and protein ubiquitination may have a regulatory role in cells under drought stress (Asaoka and Ikeda, 2015). Furthermore, the declined accumulation of translocon-associated protein (TRAP) subunit beta, which is implicated in the translocation of specific proteins and in the correction of misfolded proteins, could indicate that osmotic stress leads to a significant reduction in the efficiency of protein translocation in Gannong No.3 (Mesbah et al., 2006). Ziogas et al. (2015) found that the ribosomal proteins (60S and 40S ribosomal protein) were up-regulated in citrus response to PEG stress. However, our data found that the accumulation of four ribosomal proteins (i.e., gi|657380134, gi|357446687, gi|357438209, and gi|657377014) was repressed in Gannong No. 3 under osmotic stress. These findings collectively indicated that a negative effect of osmotic stress on stress-defense protein biosynthesis was more obvious in Gannong No. 3 than Longzhong.

Molecular chaperones are implicated in assisting with the correct folding of other proteins in the crowded molecular environment. Heat shock proteins (HSPs) are involved in protein folding, assembly, translocation and degradation and play an essential role in protecting plants against stress (Wang et al., 2004). Ma et al. (2016) have reported enhanced expression of HSPs in alfalfa leaves during salt stress. Previous studies have also found that Kunitz-type trypsin inhibitor was induced by various forms of biotic and abiotic stress (Srinivasan et al., 2009). As expected, the increased accumulation of HSP (gi|357480003) and two Kunitz-type trypsin inhibitors (gi|357498545 and gi|657399012) could be regarded as an important defensive response of Gannong No. 3 against osmotic stress. Similarly, the induction of Kunitz-type trypsin inhibitors has been reported in tobacco (Huang et al., 2010) and soybean (Mathesius et al., 2011). Furthermore, the accumulation of eukaryotic peptide chain release factor subunit 1 (eRF 1-1) was observed in Gannong No. 3, suggesting that eRF1-1 may be a key molecular switch that governs the termination of protein synthesis under osmotic stress (Buckingham et al., 1997; Joshi et al., 2016).

Osmotic stress altered the abundance of proteins related to amino acid biosynthesis. Lysine-ketoglutarate reductase (LKR) and saccharopine dehydrogenase (SDH) catalyze the two initial steps of lysine catabolism (Zhu et al., 2002). Here, the up-regulation of LKR/SDH (gi|357479289) in both alfalfa varieties suggested that lysine catabolism may be enhanced by osmotic stress. Moreover, the increased accumulation of delta-1-pyrroline-5-carboxylate synthetase 3 (gi|356668563) and delta-1-pyrroline-5-carboxylate dehydrogenase 1 (gi|357477461) in Longzhong, could be connected to the fact that proline metabolism can be tightly regulated to provide a source of nitrogen and energy under stress conditions (Deuschle et al., 2004). This result might also imply that PEG-treated Longzhong could enhance its osmotic adjustment through regulating the proline synthesis and degradation. Similarly, Yamchi et al. (2007) also found proline accumulation in drought-tolerant tobacco during osmotic stress. Asparagine synthetase (AS) is the primary enzyme involved in asparagine production (Romagni et al., 2000). Here, the up-regulation of AS in Longzhong indicated that the formation of asparagine from aspartate and glutamine may be enhanced by osmotic stress. Additionally, homoglutathione synthetase (hGSHS) can catalyze homoglutathione synthesis and functions in the stress response of legume root nodules (Moran et al., 2000). In this study, the up-regulation of hGSHS (gi|657380358) in Gannong No. 3 suggested that changes in homoglutathione synthesis could be a response strategy to osmotic stress. However, the accumulation of hydroxymethylglutaryl-CoA (HMG-CoA) synthase (gi|357481763) and phosphoglycerate dehydrogenase (PHGDH) (gi|357467711) was all repressed by osmotic stress in both alfalfa varieties. The decreased abundance of HMG-CoA synthase, an enzyme involved in the mevalonate-dependent isoprenoid biosynthesis pathway, could suggest that osmotic stress inhibited the accumulation of isoprenoid compounds in PEG-treated alfalfa (Suwanmanee et al., 2004). Meanwhile, the declined accumulation of PHGDH, which is an essential enzyme involved in the phosphoserine (PS) pathway, implied that normal root development and secondary metabolism inside alfalfa could be negatively affected by osmotic stress (Benstein et al., 2013). Taken together, osmotic stress significantly altered the balance between protein synthesis and degradation in two alfalfa varieties, and the effects of osmotic stress on protein processing and amino acid biosynthesis was more pronounced in Gannong No. 3 than in Longzhong.

### Membrane- and Transport-Related Proteins

Proteins associated with membrane and transport also play key roles in stress responses (Hao et al., 2015). Our data showed that three LZRPs related to transmembrane transport induced by osmotic stress. Multidrug and toxic compound extrusion (MATE) transporters use ion gradients (either H^+^ or Na^+^) across the membrane to drive substrate export (Morita et al., 2009). Protein tolB is specifically responsible for secreted protein translocation across the outer membrane (Cao and Klebba, 2002). Phytocyanins (PCs), containing a plastocyanin-like domain (PCLD), are plant-specific blue copper proteins participated in photosynthetic electron transport, plant development and defense responses to adverse environmental conditions (Oztur et al., 2002; Xu et al., 2017). Here, the increased accumulation of MATE subfamily protein, protein tolB, and PCLD protein in Longzhong could suggest that these proteins play important roles in ion, electron and macromolecules transport under osmotic stress. However, the accumulation of eight GNRPs related to transmembrane transport was suppressed upon osmotic stress. Here, the lower abundance of major facilitator superfamily (MFS) domain-containing protein (gi|357464047) and sugar porter (SP) family MFS transporter (gi|657381361) may result in reductions in the transportation of ions and solutes under osmotic stress (Quistgaard et al., 2016). Plasma membrane intrinsic proteins (PIPs) mediate water transport in many plant species. In this study, we observed the down-regulation of aquaporin PIP11 (gi|357492595) and aquaporin (gi|222431975) in Gannong No. 3, suggesting that osmotic stress may reduce the permeation of water across membranes. Similar results were obtained by Mahdieh et al. (2008) in *Nicotiana tabacum* under drought stress. In addition, the lower abundance of synaptotagmin-7 (gi|357438479) and a class I ADP ribosylation factor GTPase-activating protein (AGD10) (gi|357517049) in Gannong No. 3 suggested that membrane trafficking pathways could be inhibited by osmotic stress (Yoo et al., 2008). ADP/ATP carrier protein and phosphate carrier protein are responsible for the proper targeting of soluble cargo proteins and the phosphate transport, respectively (Ahmed et al., 2000; Roso and Vidal, 2010). Here, the declined abundance of ADP/ATP carrier proteins (gi|657388858) and phosphate carrier protein (gi|357508379) in Gannong No. 3 suggested that these carrier proteins may be involved in alfalfa responses to osmotic stress. All together, these results indicate that proteins involved in transmembrane transport were significantly induced in stressed Longzhong but suppressed in stressed Gannong 1No. 3.

### Signal Transduction-Related and Transcription-Related Proteins

Drought-induced cellular signal transduction involves ligands binding to membrane-associated receptors, generation of second messengers and activation of phosphorylation cascades (Hao et al., 2015; Joshi et al., 2016). In this study, osmotic stress altered the abundance of nine DAPs involved in signal transduction, including three down-regulated LZRPs and six GN3RPs (four up-regulated and two down-regulated). Monooxygenases are biocatalysts that catalyze the incorporation of one oxygen atom into organic substrates involved in many metabolic pathways (Torres Pazmiño et al., 2010). The cytochrome P450 (CYP) monooxygenases are implicated in the synthesis of secondary metabolites, especially in saponin biosynthetic pathway, and participate in maintaining the homeostasis of phytohormones (Yan et al., 2016). Previous studies have shown that the expression level of CYP716A12 is higher and more stable in roots of *Medicago truncatula* compared with leaves and stems under normal growth conditions, while its expression is suppressed in leaves of pathogen-infected plants as compared with the non-damaged control plants (Carelli et al., 2011; Martin, 2011). Our data demonstrated that two monooxygenases (gi|71534999) were induced in Gannong No. 3 but cytochrome P450 monooxygenase CYP716A12 (gi|698347596) was suppressed in Longzhong. These findings may have occurred because the accumulation of monooxygenases involved in various metabolic pathways may be differentially regulated by osmotic stress in two contrasting drought-tolerant alfalfa varieties.

Moreover, many of the accompanying stress responses are mediated by jasmonates (Verma et al., 2016). 12-oxophytodienoate reductase, the rate-limiting enzyme implicated in the biosynthesis of jasmonic acid, were induced by osmotic stress in Gannong No. 3, suggesting that jasmonic acid-associated metabolism may play a vital role in the defense signaling pathway of stressed alfalfa (Stenzel et al., 2003). Furthermore, the increased abundance of cysteine-rich RLK (receptor-like kinase) protein (gi|657399623), which is involved in direct ROS sensing and stress responses, could suggest that it plays a crucial role in the ROS signaling of Gannong No. 3 to maintain the balance between defense and growth (Idänheimo et al., 2014). Multicopper oxidases are enzymes that catalyze the oxidation of various substrates, such as metals, phenolic compounds, and the reduction of O_2_ to H_2_O, and function in lignin degradation, stress defense and morphogenesis (Butterfield et al., 2015). Here, the down-regulation of multicopper oxidases (MCOs) in Longzhong may be involved in the defense response of alfalfa to osmotic stress. Additionally, the decreased abundance of lectin (gi|2951684), involved in cellular regulation and protein–carbohydrate interactions, could indicate it also play pivotal roles in the signal transduction required for drought responses, which agrees with the findings of Jiang et al. (2010) and Sengupta et al. (2011). Purple acid phosphatases (PAPs) function in ROS generation and/or scavenge or stress-activated signal transduction. Oxidative stress and NaCl stress but sufficient phosphorus (P) enhances the expression of *GmPAP3* gene (Liao et al., 2003). In this study, the declined abundance of inactive purple acid phosphatase-like protein (gi|657394293) in Gannong No. 3 suggest this protein may participate in the stress adaptation of alfalfa. Taken together, these results suggested that signal transduction pathways function in metabolic control and adaptation to osmotic stress. The changes in accumulation of proteins linked to signal transduction was more sensitive in Gannong No. 3 compared with drought-tolerant Longzhong.

Mechanisms regulating gene expression include both transcriptional and post-transcriptional controls in most living organisms (Mazzucotelli et al., 2008). Deoxyuridine triphosphate nucleotidohydrolase (dUTPase) is a crucial enzyme that prevents uracil misincorporation during *de novo* DNA synthesis (Hogrefe et al., 2002). DNA methylation is a powerful mechanism for regulating gene expression. *o*-diphenol-*O*-methyltransferase (OMT) is essential for *de novo* DNA methylation, gene regulation and differentiation pathways in higher plants (Agorio and Vera, 2007; Butt et al., 2014). Here, the decreased abundance of dUTPase (gi|657393760) in Longzhong and OMT (gi|6688808) in Gannong No. 3 suggested that these proteins may participate in the regulation of *de novo* DNA synthesis under PEG stress. Furthermore, RNA-binding proteins (RBPs) play an important part in post-transcriptional controls through interactions with RNA, such as splicing, polyadenylation, mRNA stabilization and localization, and translation (Ciuzan et al., 2015). In this study, the lower accumulation of RBPs (gi|357464165) in Gannong No. 3 suggested that normal post-transcriptional regulation processes would be suppressed by osmotic stress. Taken together, osmotic stress had adverse effects on the DNA synthesis of both alfalfa varieties and significantly inhibited mRNA processing in Gannong No. 3.

### Cell Wall and Cell Cytoskeleton Metabolism-Related Proteins

The plant cell wall can be considered a protective barrier and is a complex cellulose-hemicellulose network embedded in a matrix of pectic polysaccharides and structural proteins (Muthurajan et al., 2011). Morphological changes in plants subjected to drought stress are thought to result from cell wall modifications (Gall et al., 2015). Our data found that osmotic stress changed the abundance of nine DAPs related to cell wall and cell cytoskeleton metabolism, including two up-regulated LZRPs, five GNRPs (one up-regulated and four down-regulated), and two up-regulated proteins shared by both varieties. Of these, xyloglucan-specific endoglucanase inhibitor protein and expansin-B1-like protein involved in cell wall modification were induced by osmotic stress in both varieties. Osmotic stress also altered the abundance of proteins related to cell wall formation and degradation. Here, the increased abundance of xyloglucan endotransglucosylase/hydrolase (XTH) (gi|357479711) could indicate that cell-wall adjustment may be a protective mechanism of Longzhong roots against water deficit. Similarly, Cho et al. (2006) have confirmed the role of XTH in improving drought tolerance. Polygalacturonase (PG) is a major pectin-degrading enzyme that catalyzes the hydrolytic cleavage of glycosidic linkages in pectic polymers. Beta-xylosidases are involved in the final decomposition of plant cell wall hemicellulose (Gall et al., 2015). Here, the increased abundance of PG-like protein (gi|357504351) in Longzhong and beta-xylosidase (gi|357449039) in Gannong No. 3 suggested that osmotic stress may result in cell wall degradation. Cytoskeleton protein regeneration is considered a major factor affecting cell cycle progression (Zhou et al., 2013). Tubulin is the subunit of cellular microfilaments and microtubules. Tubulin α and β are key components of the eukaryotic cytoskeleton (Wasteneys, 2004; Jin et al., 2016). Sengupta et al. (2011) observed the repression of tubulin proteins in roots of *Vigna radiata* during a 3-day drought stress treatment. In the present study, the declined abundance of two cytoskeleton-related proteins, including tubulin beta-1 chain (gi|657386638) and tubulin beta chain (gi|357469063), was observed in Gannong No. 3 subjected to osmotic stress. Suppression of these proteins may lead to low cell-cycle activity under drought stress. Moreover, Takác et al. (2011) proposed that profilin 2 may be engaged in the interconnection between the actin cytoskeleton and vesicular trafficking. Here, the down-regulation of cytoskeleton-related profilin 2 (gi|14423868) suggested that osmotic stress may have a negative effect on cytoskeleton dynamic reorganization in Gannong No. 3. Nevertheless, these results signify that alterations in root morphology-related proteins could be an indispensable adaptive response to osmotic stress in alfalfa.

### Unknown and Common Stress-Inducible Proteins

We identified proteins known to play critical roles in drought responses as well as predicted or unknown proteins that may have important functions in the regulatory network for osmotic stress. Our results showed that among the 1901 proteins, 169 proteins had predicted or unknown functions and 22 of those proteins exhibited an opposite expression pattern in the two alfalfa varieties, indicating cultivar-specific functions under drought stress. Moreover, three proteins (i.e., gi|502122510, gi|571481908, and gi|502176380) were found to be expressed in a similar pattern between Longzhong and Gannong No. 3, suggesting that there are also common stress responses in the two varieties. Those stress-responsive proteins with predicted functions may also confer drought tolerance. Therefore, further studies of these proteins will help elucidate the molecular mechanisms behind osmotic stress responses of alfalfa varieties differing in drought tolerance.

## Conclusion

**FIGURE 8:**
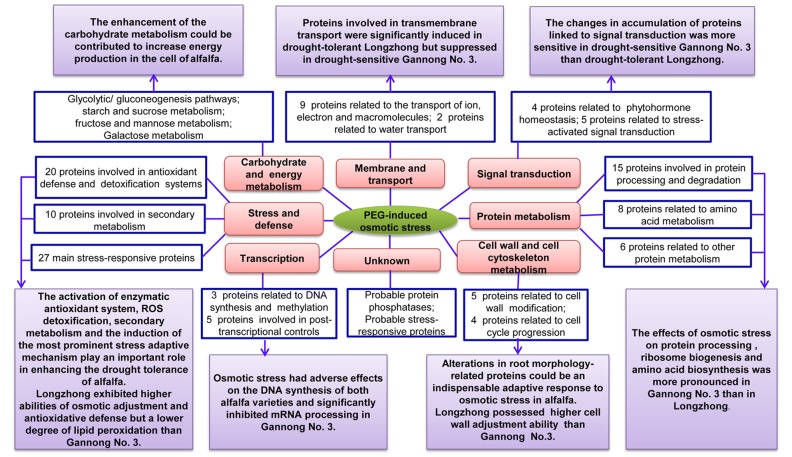
Model shows the differential responses of two alfalfa varieties with contrasting drought tolerance to PEG-induced osmotic stress based on physiological and proteomic changes.

In this study, we conducted a comprehensive comparative analysis of two alfalfa varieties contrasting in drought tolerance based on their physiological and proteomic changes. Our results demonstrated that Longzhong was more tolerant to osmotic stress than Gannong No. 3, which was evidenced by quantitative and qualitative differences at the physiological and proteomic levels. Longzhong exhibited higher abilities of osmotic adjustment and antioxidative defense but a lower degree of lipid peroxidation than those of Gannong No. 3 under stress conditions. A total of 142 DAPs including 52 LZRPs, 71 GN3RPs, and 19 proteins shared by two varieties were identified using iTRAQ. The proteomic changes suggested more significant metabolic flexibility in Longzhong than in Gannong No. 3 roots, thus enhancing the drought tolerance of Longzhong. Transcriptional expression analysis showed that the expression levels of nine genes (*MtALDH*, *MtPFK*, *MtMLP*, *MtPLPP*, *MtLKR*, *MtEBLP, MtGI, MtHMCS*, and *MtGTF*) were corresponded well with the DAPs expression changes. A model of comparative physiological and proteomic analysis of the two alfalfa varieties’ differential responses to PEG-induced osmotic stress is shown in **Figure 8**. The higher drought tolerance of Longzhong may be attributed to the following factors: (a) higher osmotic adjustment capacity, (b) greater coordination of its enzymatic and non-enzymatic antioxidant systems, (c) early activation of stress defense and detoxification, (d) maintenance of a better balance between protein processing and degradation, (e) promoting the trans-membrane transport of ion, electron, and protein, and (f) improved cell wall adjustment capacity. These results may improve the understanding of the physiological and molecular mechanisms of alfalfa adaptation or tolerance to drought stress. Further studies directed toward integrating morpho-physiological analysis, molecular biology, along with the –omics approaches are needed to elucidate the complex regulatory networks of alfalfa drought response and to identify core genes and proteins. The genetic manipulation of these candidates will substantially accelerate the breeding progress of developing novel drought-tolerant alfalfa varieties and improve their economic yield under adverse environmental conditions.

## Author Contributions

CZ and SS initiated and designed the experiments. SS guided the research. CZ performed the experiments, analyzed the data, and wrote the manuscript. All authors have read and approved the final manuscript.

## Conflict of Interest Statement

The authors declare that the research was conducted in the absence of any commercial or financial relationships that could be construed as a potential conflict of interest.

## Footnotes

1 www.iprox.org

2 http://www.geneontology.org

3 www.genome.jp/kegg/

4 http://primer3.ut.ee/

## Abbreviations

2-DE: two-dimensional gel electrophoresis
ACN: acetonitrile, APX, ascorbate peroxidase, AsA, reduced ascorbate, AsA/DHA, the ratio of reduced to oxidized ascorbate
CAT: catalase
DAPs: Differentially accumulated proteins
DHA: oxidized ascorbate
DTT: dithiothreitol
FA: formic acid
GN3RPs: Gannong No. 3-specific, drought responsive proteins
GO: Gene Ontology
GR: glutathione reductase
GSH: reduced glutathione
GSH/GSSG: the ratio of reduced to oxidized glutathione
GSH-Px: glutathione peroxidase
GSSG: oxidized glutathione
GST: glutathione-S-transferase
H_2_O_2_: hydrogen peroxide
HPLC: high performance liquid chromatography
iTRAQ: isobaric tags for relative and absolute quantification
KEGG: Kyoto Encyclopedia of Genes and Genomes
LC-MS: liquid chromatograph-mass spectrometer
LEA: late embryogenesis abundant
LZRPs: Longzhong-specific, drought responsive proteins
MDA: malondialdehyde
O_2_^•-^: superoxide anion free radical
OH^•^: hydroxyl free radical
PAGE: polyacrylamide gel electrophoresis
PEG: polyethylene glycol
POD: peroxidase
PPFD: photosynthetic photon flux density
ROS: reactive oxygen species
RT-qPCR: real-time quantitative polymerase chain reaction
SDS: sodium dodecyl sulfate
SOD: superoxide dismutase
T-AOC: total antioxidant capability
TBA: 2-thiobarbituric acid
TCA: trichloroacetic acid
TEAB: triethylammonium bicarbonate
TTC: triphenyl tetrazolium chloride
