# Supplementary material for: Physiological and Proteomic Responses of Contrasting Alfalfa (Medicago sativa L.) Varieties to PEG-Induced Osmotic Stress
Source: Front Plant Sci. 2018 Feb 28;9:242. doi: 10.3389/fpls.2018.00242 (PMC5835757; doi:10.3389/fpls.2018.00242)
Supplement: Supplementary file 1 [file Presentation_1.PDF]

Supplementary Material

Physiological and Proteomic Responses of Contrasting Alfalfa (*Medicago sativa* L.) Varieties to PEG-induced Osmotic Stress

Cuimei Zhang, Shangli Shi*

***Correspondence:**
Prof. Shangli Shi
shishl@gsau.edu.cn

1 Supplementary Figures

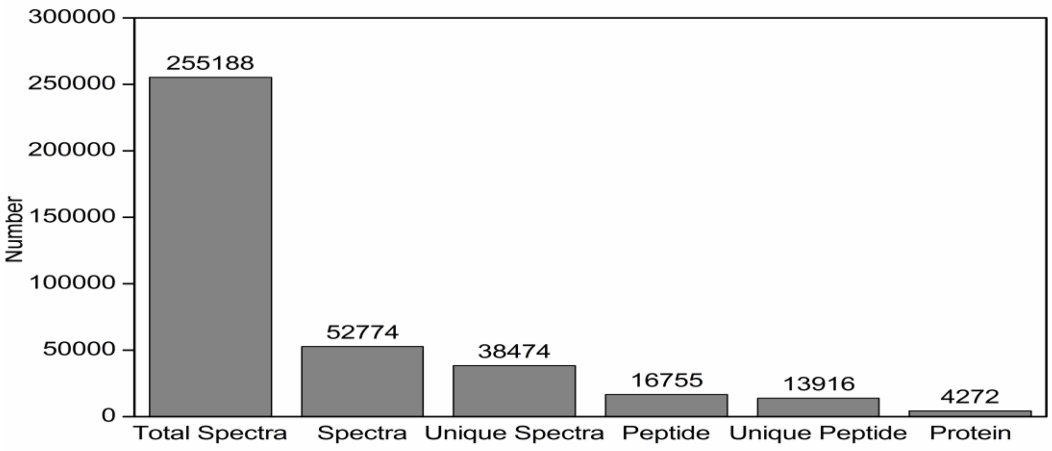

Supplementary Figure 1. Spectra, peptides and proteins identified from iTRAQ proteomics after searching against the sequence databases.

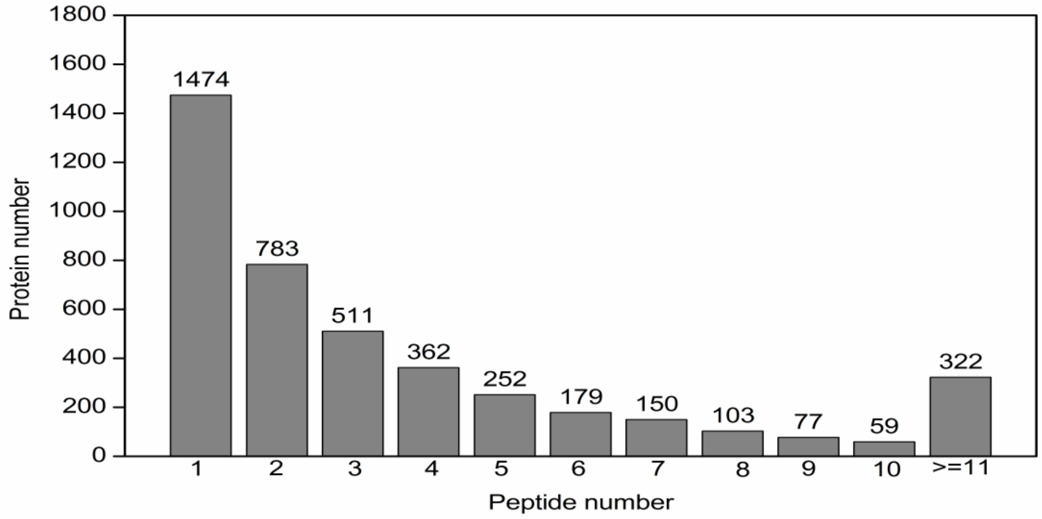

Supplementary Figure 2. Number of peptides that were matched to proteins using MASCOT.

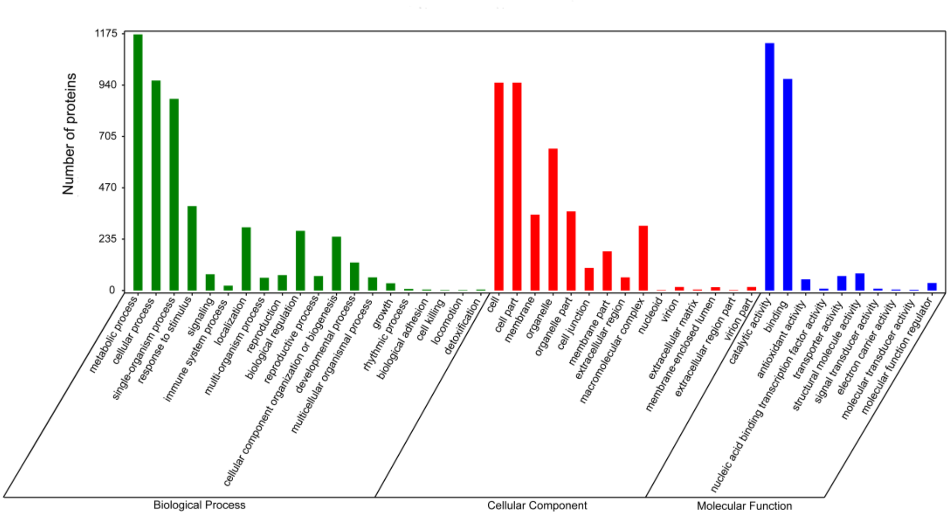

Supplementary Figure 3. Gene Ontology (GO) analysis of the all identified proteins in roots of two alfalfa varieties under PEG-induced osmotic stress.

2 Supplementary Data

The Legends for Supplementary Data:

Supplementary Data Sheet 1. Quantitative information of all identified proteins in the first biological replicate of this study

Supplementary Data Sheet 2. Quantitative information of all identified proteins in the second biological replicate of this study

Supplementary Data Sheet 3. Quantitative information of all identified proteins in the third biological replicate of this study

Supplementary Data Sheet 4. All identified proteins in roots of two alfalfa varieties under PEG-induced osmotic stress.

Supplementary Data Sheet 5. Differentially accumulated proteins (DAPs) in *Medicago sativa* L.cv.Longzhong roots subject to PEG-induced osmotic stress.

Supplementary Data Sheet 6. Differentially accumulated proteins (DAPs) in *Medicago sativa* L.cv. Gannong No.3 roots subject to PEG-induced osmotic stress.
